# Improving Advance Care Planning for Hospitalized Patients With Heart Failure

**DOI:** 10.1089/pmr.2023.0035

**Published:** 2023-12-20

**Authors:** Tobin Mathew, Akash Patel, Kyle DiGrande, Nathalie De Michelis, Behram Mody, Dawn Lombardo

**Affiliations:** Division of Cardiology, Department of Medicine, University of California, Irvine Medical Center, Orange, California, USA.

**Keywords:** advanced directives, electronic medical record, heart failure

## Abstract

Advance care planning (ACP) is a valuable and proven approach for enhancing end-of-life communication and quality of life for individuals with heart failure (HF) and their family members. However, the adoption of ACP in practice is still lower than desired. According to University of California, Irvine Medical Center HF metrics, only 15.3% of hospitalized HF patients had completed ACP documentation before discharge, as recorded in the electronic medical record (EMR). This quality improvement project aimed to investigate whether the rate of ACP completion could be increased by utilizing EMR reminders to health care teams regarding individual patients. Personalized reminders were sent to providers for each hospitalized patient diagnosed with HF, who did not have existing ACP documentation in the EMR, to encourage completion of ACP documentation. Our findings have shown that, during the three-month intervention period, the average ACP completion rate was 21.0%. This represents a 5.7% absolute increase in ACP completion compared to the six months before our intervention (15.3%); a relative increase of 37.3%. Direct message reminders to providers prove to be an effective method for enhancing ACP completion among this specific patient group.

## Objectives

- Discuss factors affecting advance care planning (ACP) documentation.
- Implement direct communication with inpatient care teams for patients with the diagnosis of heart failure to perform ACP documentation before discharge.
- Determine the percentage of completed ACP during the intervention period and compare it to ACP completion rates during periods outside the intervention timeframe.
- Discuss implications of intervention and future steps to improve ACP based on results.

## Introduction

Heart failure (HF) is a condition associated with significant mortality, causing patients to undergo a gradual decline in quality of life. There have been improvements in management, resulting in an increased number of patients living with symptomatic and progressive HF to cardiogenic shock and death. Therefore, it is important for HF patients to establish advanced care planning (ACP).^1^ According to the Delphi panel, ACP can be defined as a “process that supports adults at any age or stage of health in understanding and sharing their personal values, life goals, and preferences regarding future medical care.”^2^ An advanced directive (AD) is a commonly used formalized legal document designed to communicate such values. ACP is an important and proven strategy to improve end-of-life communication and quality of life of patients with HF.^3^

**FIG. 1.**
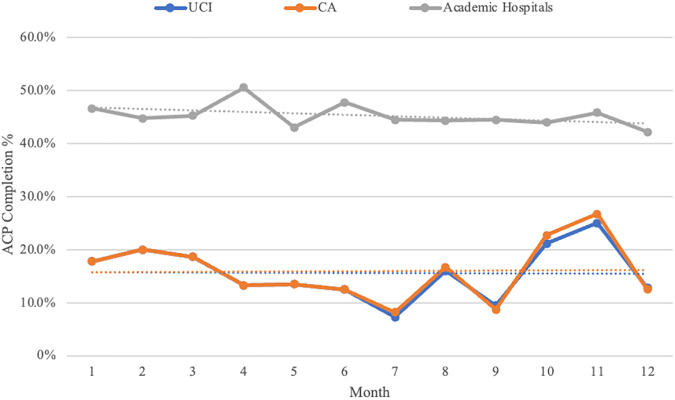
ACP rate from UCI compared with average rate among California hospitals and the national average rate among academic medical centers through 2021. Associated trendlines are depicted. Data are taken from the Advanced Certification in HF metrics.^4^ ACP, advance care planning; CA, California; UCI, University of California, Irvine Medical Center.

Unfortunately, the frequency of ACP conversations in practice remains low. Advanced Certification in Heart Failure (ACHF) metrics show that in 2021, only 15.9% of hospitalized HF patients in California had completed ADs at discharge.^4^ Our institution, which is an advanced HF medical center with a dedicated HF service and Left Ventricular Assist Device implantation capabilities, has similarly low rates of ACP completion. As seen in Figure 1, University of California, Irvine Medical Center (UCI) HF metrics show that between July 2021 and December 2021, an average of 15.28% of HF patients hospitalized at UCI had completed ACP at discharge, as measured by presence of Physician Orders for Life-Sustaining Treatment form or AD in the electronic medical records (EMRs). The average percentage for all of 2021 at UCI was 15.6%, like that seen at other California hospitals.^4^ However, academic hospitals in the United States had an average of 45.2% ACP completion rate in 2021 per ACHF metrics. It is unclear why California hospital ACP documentation rates lag academic hospitals nationwide. The goal of this quality improvement project was to improve ACP completion percentage at hospital discharge using EMR reminders to the health care team.

## Methods

ACP completion percentages were obtained from analyzing internal data from our EMR collected by UCI's Advanced HF Quality Task Force. Each month, the HF department receives data on presence of ACP documentation among patients with the diagnosis of HF.

For a three-month period (January 2022–March 2022), our research team messaged providers—which consisted of physicians (including medical residents and fellows), physician associates, and nurse practitioners—for hospitalized patients with a diagnosis of HF without existing ACP documentation in the EMR. Patients who were coded in the EMR as having a HF diagnosis during the hospital stay, regardless of type, acuity, or severity, were identified as possible candidates, without exclusion. No additional patient data were collected such as demographics or sample characteristics. For each patient included, ACP documentation was viewed as reported on the EMR; if such documentation was absent, they were selected for intervention. Messages were sent via EMR's secure chat function, serving as a reminder to complete ACP documentation. On the day of admission, an initial notification was dispatched to the provider who was designated in the EMR as the primary provider assigned to the identified patient for that day. Most commonly, this provider was the medical resident of the admitting internal medicine team. A follow-up message was sent to the designated primary provider two weeks after admission date if the patient continued to be hospitalized without ACP completion. ACP documentation completion percentage was calculated by our team using the EMR data during the three-month study period as our experimental group. This was then compared to the completion percentages for UCI as listed from the 2021 ACHF metrics, which served as our control.

## Results

During the three-month intervention period, we found that the average completion rate of ACP documentation at UCI was 21.0%. This represents a 5.7% absolute increase in ACP completion compared to the six-month period before our intervention (15.3%), which equates to a relative increase of 37.3%. Table 1 reflects the collected data over the aforementioned time frames. Furthermore, this corresponds to a 5.4% increase from the average annual ACP completion rate at UCI in 2021 (15.6%), reflecting a relative increase of 34.4% during our three-month intervention period. There were 157 patients included in the initial study, with an increase of 9 patients having ACP documentation after intervention. The run-line change was not significant and not demonstrated.

**Table 1.** Advance Care Planning Completion Rates for Patients With Heart Failure at University of California, Irvine Medical Center in the 12 and 6 Months Before Intervention, and the 3-Month Intervention Period Completion Rates

	Preintervention period (12 months)	Preintervention period (6 months)	Intervention period (3 months)
ACP completion rates (%)	15.6	15.3	21.0

ACP, advance care planning.

## Discussion

In this quality improvement intervention, EMR-based reminders sent to the primary providers of hospitalized HF patients increased the rate of ACP documentation before discharge. In the six months before this intervention, only 15.3% of hospitalized HF patients had documented ACP. During the intervention, there was an improvement from 15.3% to 21.0% compared with a representative six-month sample during 2021. This represents a 37.3% relative increase in completion percentage compared with the control group average over the previous six months to the intervention. Additionally, the completion percentage for all of 2021 was 15.6%; our intervention improves on this metric by 5.4%; a 34.4% relative increase. This result suggests that our intervention can provide a simple method to promote ACP discussions in hospitalized HF patients.

ACP has been shown to provide numerous benefits for patients and their families, including improved quality of life as well as increased satisfaction with end-of-life care and communication.^3^ It also benefits the health care system by reducing health care costs and lowering in-hospital deaths.^5^ However, nationwide participation in ACP continues to be a challenge, with estimates suggesting that <50% of adults with serious illnesses have completed an AD.^6^ Reasons for this include the time-consuming nature of such discussions and the lack of patient and provider familiarity and discomfort.^7^ Approaches to improving ACP have typically involved personalized interactions to facilitate patient education about HF. However, interventions focused solely on patient education have demonstrated limited effectiveness in promoting ACP.^8^

ACP is relevant for HF patients given high mortality rates, symptom burden, and health care costs.^9,10^ HF guidelines recommend ACP documentation as part of standard of care.^11^ Nevertheless, there is a notable disparity in ACP for HF compared to other serious illnesses, like cancer. Interventions to improve ACP documentation in this specific population have shown that patient-mediated interventions, reminder systems, and educational meetings were all effective at increasing ACP completion, particularly when used in combination.^12^ These studies conducted faced certain limitations, as implementing such approaches would be time-consuming and resource-intensive.

The main strengths of our intervention are its simplicity and practicality. A personalized EMR message reminded the primary providers at regular intervals to prioritize ACP discussions with their HF patients. Previous studies examining ACP in non-HF populations have demonstrated the effectiveness of EMR reminders to increase ACP documentation.^13^ The primary care teams were not placed under time constraints, as opposed to the outpatient setting. Additionally, conducting these discussions within an academic setting offers the advantage of having physician trainees available to participate, which contributes to their education and emphasizes the importance of ACP to these future physicians.

Limitations of our intervention include the duration of our intervention as three months, which could pose difficulties in generalizing the findings due to potential fluctuations in hospitalization patterns throughout the year, influenced by a range of factors such as seasonal changes, economic shifts, and societal trends. The inclusion criteria also do not differentiate patients who are presenting to the hospital with HF versus a separate diagnosis with a history of HF. Additionally, there is no designation of severity of HF or type of HF (e.g., diastolic vs. systolic HF). However, the ACHF collects the data in a similar fashion. Our control group may have been influenced by the COVID-19 pandemic, with unclear specific impact on ACP completion. An additional source of bias that limits direct comparison of the experimental and control groups is that the ACP completion percentage was measured by the research team in the experimental group, while the control group consisted of the ACP completion percentage from the ACHF data for UCI. It is also important to note that the sustainability of the reminders and their effectiveness across different hospital settings cannot be determined from this study alone. Furthermore, we acknowledge the potential for alarm fatigue with this intervention if it were implemented longitudinally, as providers may become desensitized to an additional alert with a request for a potentially time-consuming intervention during a hospital stay. The intervention's impact therefore may decrease over time and its priority should be weighed against other important interventions that are needed during a busy hospital stay. Further interventions of this kind could attempt to address this limitation by changing the timing of the initial alert to be later in a hospital course, so that its consideration is not competing with as many variables associated with the initial admitting stages of a hospitalization. To draw more comprehensive and applicable conclusions, larger interventional cohorts should be utilized. Additionally, further longitudinal analysis of quality of metrics in association with ACP would lend additional support to the impact of interventions targeted at ACP completion.

## Conclusion

ACP among hospitalized HF patients remains low. In our quality improvement study, direct message reminders via EMR to providers have shown to improve ACP completion in HF inpatients before discharge. Implementation of systemwide communication to providers regarding ACP may serve as a means of improving ACP completion among this subset of patients.

## Funding Information

No funding was received.

## Abbreviations Used

ACHF: Advanced Certification in Heart Failure
ACP: advance care planning
AD: advanced directive
EMR: electronic medical record
HF: heart failure
UCI: University of California, Irvine Medical Center
